# Random Forest Analysis of Out-of-Pocket Health Expenditures Associated with Cardiometabolic Diseases, Lifestyle, Lipid Profile, and Genetic Information in São Paulo, Brazil

**DOI:** 10.3390/healthcare12222275

**Published:** 2024-11-14

**Authors:** Jean Michel R. S. Leite, Lucas A. I. Trindade, Jaqueline L. Pereira, Camila A. de Souza, Júlia M. Pavan Soler, Regina C. Mingroni-Netto, Regina M. Fisberg, Marcelo M. Rogero, Flavia M. Sarti

**Affiliations:** 1 School of Public Health, University of São Paulo, São Paulo 01246-904, Brazil; lucas.akio.trindade@usp.br (L.A.I.T.); jaque.lps@gmail.com (J.L.P.); rfisberg@usp.br (R.M.F.); mmrogero@usp.br (M.M.R.); 2 School of Arts, Sciences and Humanities, University of São Paulo, São Paulo 03828-000, Brazil; flamori@usp.br; 3 Institute of Mathematics and Statistics, University of São Paulo, São Paulo 05508-090, Brazil; milaalvesdesouza@gmail.com (C.A.d.S.); pavan@ime.usp.br (J.M.P.S.); 4 Institute of Biosciences, University of São Paulo, São Paulo 05508-090, Brazil; renetto@ib.usp.br

**Keywords:** out-of-pocket healthcare expenditures, dyslipidemia, genetic risk score, GWAS, random forest

## Abstract

**Background/Objectives:** There is a lack of empirical studies of out-of-pocket health expenditures associated with dyslipidemias, which are major cardiovascular risk factors, especially in underrepresented admixed populations. The study investigates associations of health costs with lipid traits, GWAS-derived genetic risk scores (GRSs), and other cardiometabolic risk factors. **Methods:** Data from the observational cross-sectional 2015 ISA-Nutrition comprised lifestyle, environmental factors, socioeconomic and demographic variables, and biochemical and genetic markers related to the occurrence of cardiometabolic diseases. GWAS-derived genetic risk scores were estimated from SNPs previously associated with lipid traits. There was phenotypic and genetic information available for 490 independent individuals, which was used as inputs for random forests and logistic regression to explain private quantitative and categorical health costs. **Results:** There were significant correlations between GRSs and their respective lipid phenotypes. The main relevant variables across techniques and outcome variables comprised income per capita, principal components of ancestry, diet quality, global physical activity, inflammatory and lipid markers, and LDL-c GRS and non-HDL-c GRS. The area under the ROC curve (AUC) of quartile-based categorical health expenditure without GRSs was 0.76. GRSs were not significant for this categorical outcome. **Conclusions:** We present an original contribution to the investigation of determinants of private health expenditures in a highly admixed population, providing insights on associations between genetic and socioeconomic dimensions of health in Brazil. Ancestry information was also among the main factors contributing to health expenses, providing a novel view of the role of genetic ancestry on cardiometabolic risk factors and its potential impact on health costs.

## 1. Introduction

Cardiovascular diseases (CVDs) are among the major causes of mortality worldwide, resulting in premature deaths, loss of quality of life, and a substantial socioeconomic toll on individuals, communities, and national health systems [1]. For instance, the burden of CVDs in the USA has been estimated to be approximately USD 820 billion by 2030 [2].

Dyslipidemias, characterized by alterations in lipid concentrations, are significant risk factors associated with the occurrence of CVDs, primarily due to their involvement in the pathophysiology of atherosclerosis [3,4]. CVDs are not only highly prevalent in several countries, with low awareness, control, and treatment rates, but also result in a substantial burden to national health systems [5,6,7].

In the context of the Brazilian health system, there is a lack of empirical studies addressing the healthcare costs associated with dyslipidemias. A major part of the economic literature focuses primarily on analyzing healthcare costs and the economic burden of other morbidities, e.g., obesity and associated risk factors, which also comprise conditions related to CVDs [8,9]. Changes in modifiable risk factors, encompassing lifestyle characteristics, may reduce the impacts of dyslipidemias in societies worldwide [10,11].

In addition, further evidence on the role of additional risk factors linked to genetic variation has been extensively demonstrated by candidate gene, heritability, and genome-wide association studies (GWASs) employing single-nucleotide polymorphisms (SNPs) underlying lipid profiles and dyslipidemias [12,13,14,15,16,17]. Importantly, previous investigations have shown the relationship between genetic variation and healthcare outcomes [18,19]. For instance, positive associations between body mass index (BMI), polygenic risk score, and health expenditure were found [18]. Yet, there is an absence of studies on specific pathways and effects of each of these genetic markers on lipid traits and associated healthcare costs. This gap becomes even deeper when considering that populations of admixed ancestries are often underrepresented in these investigations.

Recently, in the context of the Brazilian population, several novel loci were found to be significantly associated with serum lipid traits by a genome-wide association study (GWAS) [17]. However, the extent to which these associations might have an ultimate impact on health costs linked to alterations in the lipid profile remains an unexplored question. Therefore, we aimed to perform an exploratory study to evaluate the relationship between out-of-pocket (OOP) health expenditures, lipid traits, GWAS-derived genetic risk scores, genetic ancestry, and other common cardiometabolic risk factors in a highly admixed population from Brazil. The main hypothesis is that lipid profile is associated with OOP healthcare expenditures and that using genetic variation significantly associated with lipids through genetic risk scores provides a better understanding of these costs. 

## 2. Material and Methods

### 2.1. Study Design and Population

This investigation used data from the observational cross-sectional survey “2015 Health Survey of São Paulo with Focus on Nutrition (2015 ISA-Nutrition)”, a population-based study with a probabilistic sample of individuals living in the urban area of São Paulo (SP). São Paulo is the largest city in South America and Brazil, and it has the greatest contribution to the country’s Gross Domestic Product (GDP) according to the Brazilian Institute of Geography and Statistics (IBGE). The 2015 ISA-Nutrition aimed at the evaluation of lifestyle and environmental modifiable factors, socioeconomic and demographic variables, and biochemical and genetic markers related to the occurrence of cardiometabolic diseases. It was approved by the Research Ethics Committee of the School of Public Health from the University of São Paulo (#43838621.7.0000.5421 and #30848914.7.0000.5421 for the 2015 ISA-Nutrition). Previously from data collection, informed written consent was appropriately obtained from 2015 ISA-Nutrition participants. Further details on the study design and data collection procedures can be found elsewhere [20].

Individuals included in the present study were selected based on two-stage cluster sampling stratified by conglomerates (urban census tracts and households), to ensure representativeness at the population level. Participants were interviewed in their households by trained researchers in 2015, using structured questionnaires within the ISA-Nutrition survey to obtain comprehensive information on demographic, socioeconomic, lifestyle, and other characteristics. Adopting the criteria set forth by both the World Health Organization (WHO) and the Brazilian Statute for Children and Adolescents, participants were categorized into three age groups: adolescents were defined as individuals aged 12 to 19, adults as those aged 20 to 59, and the elderly as individuals aged 60 years and older. A subsample of 901 participants was randomly selected for the collection of venous blood samples allowing the assessment of biochemical markers, nutritional status, and genetic biomarkers. After the exclusion of individuals with familial relatedness and missing data, biochemical, genotypic, sociodemographic, economic, and lifestyle information was available for 667 individuals.

### 2.2. Healthcare Expenditure and Socioeconomic and Demographic Variables

Household income per capita in adult equivalents, used for fractional classification of individuals of the sample according to their socioeconomic status in the population, was estimated through the calculation of adult equivalents in the household with the following equation:

e_h_ = (Ah *ϕ* + Kh)* ^θ^*

Ah is the number of adult individuals (older than 14 years of age) in the household h; and Kh is the number of individuals aged lesser than 14 years in the household. The parameters *ϕ* and *θ* are set at 0.75, according to Deaton’s original weight proposal (1997) [21], to avoid underestimation of income effects based on the adoption of household income per capita, due to the lower requirements attributable to younger individuals in the domestic unit.

Herein, we evaluated OOP healthcare expenditures, also using the terminology “private healthcare costs/expenditures” or “private costs/expenditures” for convenience. Thus, the private healthcare expenditure variable refers to the disbursements self-reported by individuals on medical consultations, consultations with other healthcare professionals, medications, hospitalizations, tests, orthotics and prosthetics, dental consultations, private health insurance, and other healthcare expenses. The values were self-reported for the last 30 days prior to the interview and summed to obtain the total amount spent on healthcare.

The variable corresponding to private healthcare expenditures was also transformed into natural logarithm to allow comparison with other indicators and converted into tertiles and quartiles to allow estimation of logistic models. We used both tertiles and quartiles to have a more comprehensive measure of health expenditures. The economic variables evaluated are described as follows:

- Natural logarithm transformation—Due to zero inflation (a considerable number of individuals had no expenses associated with private health services), 0.001 were added to all original values of the variable so as to perform logarithm transformations. Importantly, throughout the study, we refer to “original variable” as the one with the extra 0.001.
- Logarithm with base 10.
- Two categories considering quartile distribution, i.e., values above the 2nd quartile (median) were defined as high expenditure, while values equal to or below the 2nd quartile were defined as low.
- Two categories considering its tertile distribution (combining 1st and 2nd tertiles in one category named low cost and the 3rd tertile considered high cost).
- Normalization of OOP costs by maximal and minimal values using the formula, Ny = (Oy − min(Oy)/(max(Oy) − min(Oy)); where Ny corresponds to the normalized health expenditure, Oy to the original health expenditure, and min(Oy) and max(Oy) correspond to the minimum and maximum values of the original health expenditure, respectively.

Other socioeconomic and demographic variables included sex, age, age group, ethnicity, and educational attainment. The age group (adolescents, adults, and older adults) was defined as mentioned in the previous section. Ethnicity (Black, White, Asian, Brown/Pardo, Indigenous, and others) was self-reported according to the Brazilian Census categories. Education attainment was measured using an adequacy score ranging from 0 to 1, which accounted for the expected years of education considering the individual’s age. The score was calculated by comparing the self-declared education level to the expected level of education for the individual’s age, considering the entry age in the Brazilian educational system. Scores closer to 1 represent individuals with higher educational attainment per age, and scores closer to 0 represent individuals with lower educational attainment per age.

### 2.3. Biological and Cardiometabolic Characteristics

We used comprehensive information on variables belonging to one of the following clusters:

- Inflammation: inflammatory biomarkers such as interleukin (IL)-1β, IL-6, IL-10, C-reactive protein (CRP), monocyte chemoattractant protein 1 (MCP-1), and tumor necrosis factor-alpha (TNF-α).
- Glucose metabolism: insulin, fasting blood glucose concentrations, and absence or presence of insulin resistance according to the homeostasis model assessment of insulin resistance (HOMA-IR) [22].
- Anthropometry: body mass index (BMI), waist circumference, and waist circumference to height ratio). Individuals were categorized into presence or absence of overweight (including overweight [BMI ≥ 25–29.9] and obesity [BMI ≥ 30]) using BMI, according to age group.
- Cardiovascular: systolic and diastolic blood pressure.
- Lifestyle characteristics (alcohol and tobacco use, diet quality, and physical activity).
- Lipid profile: serum concentrations of HDL-c, LDL-c, TGL, HDL-c/LDL-c, total cholesterol, VLDL-c, and non-HDL-c.

Diet quality was measured by the Revised Brazilian Healthy Eating Index (BHEI-R), which is derived from twelve dietary components that comprise total and whole fruit, total vegetables, dark green and orange vegetables and legumes, whole grains, total grains, milk, and dairy products, meats, eggs and legumes, oils, saturated fat, sodium, and a component related to the consumption of total calories from solid fat, alcohol and added sugar (SoFAAS). More information on the assessment of these components and other variables is described elsewhere [16,20,23].

### 2.4. Genetic Data and Risk Scores

Using data from a recent GWAS of quantitative lipid traits performed in the same cohort [17], we derived genetic scores that are linear combinations between the significant SNP effects and their respective genotypes. Genotype information for each marker was codified as 0, 1 and 2 for the presence of their respective minor allele and used as a discrete variable in the GWAS association analysis. Genotype calling was previously performed using Axiom™ 2.0 Precision Medicine Research Array in Thermo Fisher Scientific Laboratory (Affymetrix Inc., Santa Clara, CA, USA) [24]. The GWAS was performed using the genetic information of 330,656 SNPs for 667 unrelated individuals, with Hardy–Weinberg Equilibrium (P) ≥ 10–5 and MAF > 0.05 filters.

Hence, for each of the seven lipid traits of interest, there were genetic scores available for further association analysis (Supplementary Table S1). For more details on sample collection, DNA extraction, quantification, and quality control, and GWAS design, refer to Leite et al. [17].

### 2.5. Descriptive Statistics and Modeling of Healthcare Costs

The variables included in the investigation were used exactly as described in the previous subsections, except for the lipid traits. Particularly, since the genetic data to be used in this investigation were derived from models whose response variables (lipid traits) had to be transformed to meet statistical modeling assumptions, we employed the transformed version of these traits throughout the analysis. The Rank-Based Normal Inverse Transformation was the one chosen because it has been often and suitably used for modeling approaches that include genetic variants [16,17,25,26,27].

Since most other variables did not have a normal distribution according to the Shapiro–Wilk test, continuous variables were presented in median and interquartile range. Categorical variables were presented in frequency and 95% confidence interval for proportions.

Several models were tested according to the nature of the outcome variable, i.e., health expenditure. These models included linear regression and generalized linear model with gamma link function, and the zero-inflated quantile approach, for quantitative variables. A logistic regression model for both the quartile- and tertile-derived categorical private health cost variables was used, and its accuracy was evaluated by the area under the ROC curve (AUC).

Models were adjusted for all the covariates belonging to the six non-genetic clusters mentioned previously, including the 1st and 2nd principal components of ancestry as well as the genetic risk scores derived from the GWAS results. The first two principal components of ancestry were used for controlling population stratification along with other important base covariates such as sex, age, and body mass index (BMI). For variable selection, the Stepwise method was used, and significance was set at *α* = 0.05. Model diagnostics of linear models was performed using the Shapiro–Wilks test and the Breusch–Pagan test for normality and homoscedasticity, respectively. 

Furthermore, we employed the machine learning technique random forest (RF) as a second tool to identify genetic and non-genetic variables implicated in health costs. The number of trees in the RFs was set to 1000, and the relevance of each variable was evaluated by either the percentage increase in mean squared error (MSE) for quantitative response variables, or the decrease in Gini index for categorical variables. The former measure corresponds to the increase in the mean squared error (i.e., the mean squared difference between the observed and the predicted outcome across all trees) due to the exclusion of a given variable. The latter describes the decrease in node impurity (Gini index), which measures the proportion of misclassified observations. A lower Gini index indicates more accurate classification, i.e., the inclusion of more relevant variables leads to a higher decrease in the Gini index.

**Figure 1:**
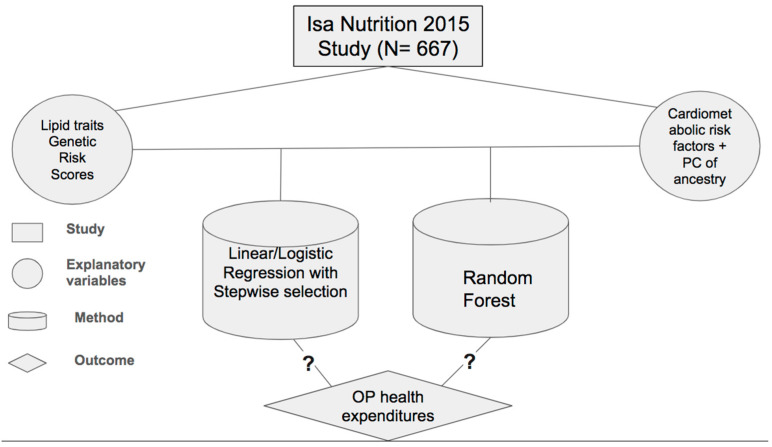
Study framework presenting data set, explanatory variables, method, and outcome under investigation, inquiring whether there are associations between the analyzed variables.

All analyses, graphs, and tables were performed and generated with R 4.3.0, and the significance level was set at 0.05. A visual depiction of the study framework is provided in Figure 1.

## 3. Results

### 3.1. Descriptive Statistics and Correlation Between Lipid Traits and GWAS-Derived Genetic Risk Scores

In order to provide initial insights on the out-of-pocket health expenditure and prevalences of major cardiometabolic risk factors, the descriptive statistics of the data set were calculated and presented in Table 1. The median value of OOP health expenditure and household income were BRL 154.00 and BRL 828.00, respectively. Overall, 49% and 33% of the sample had high health expenditures based on quartile and tertiles, respectively. There were moderate-to-high prevalences of major cardiometabolic risk factors such as dyslipidemia, insulin resistance, and overweight.

**Figure 2:**
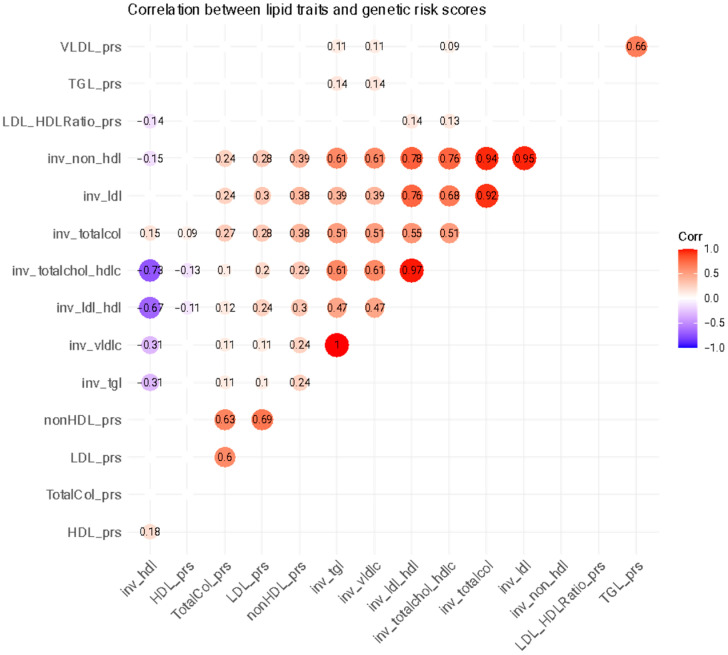
Heatmap of correlations between lipid traits and genetic risk factors. Empty and transparent circles correspond to non-significant correlation coefficients. TGL = triglycerides; LDL = low-density lipoprotein cholesterol; HDL = high-density lipoprotein cholesterol; VLDL = very-low-density lipoprotein cholesterol; prs = polygenic risk score; inv = normal-inverse-transformed version of the variable.

There were significant correlations between the lipid traits and their respective GRSs, with higher values especially for LDL-c and non-HDL-c (Figure 2).

### 3.2. Quantitative OOP Health Expenditures

**Figure 3:**
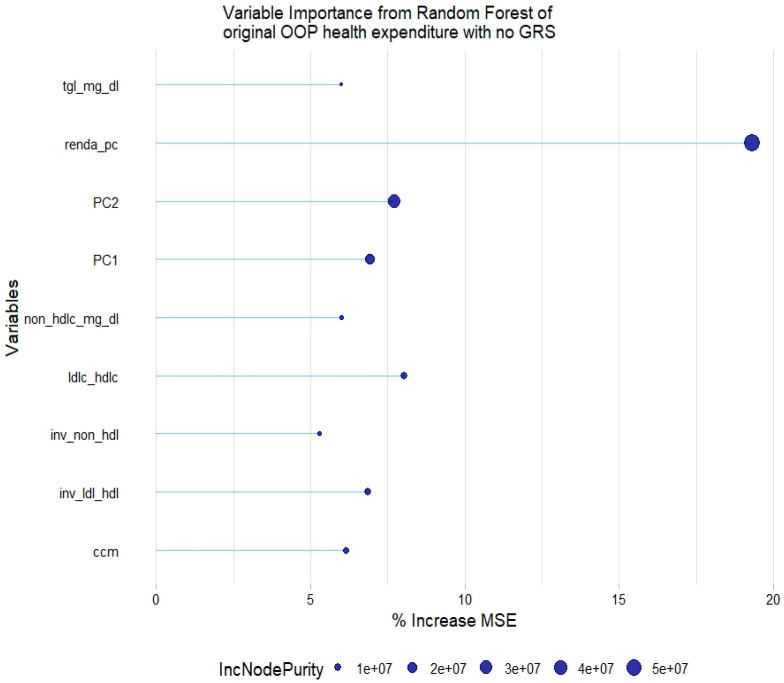
Relevant variables with more than 5% increase in mean squared error (MSE) in a random forest of original health expenditure without lipid genetic risk scores (GRSs). tgl = triglycerides; ldl-c = low-density lipoprotein cholesterol; hdl-c = high-density lipoprotein cholesterol; ldl_hdl = ldl-c/hdl-c ratio; PC = principal component of ancestry; renda_pc = household income; ccm = waist circumference; inv = normal-inverse-transformed version of the variable.

Linear regression and generalized linear regression models for quantitative variables could not be evaluated since the model assumptions were not met for any of the employed variable transformations, according to the diagnostics tests. Nonetheless, quantitative variables were successfully modeled through random forest regression. In relation to the original health expenditure variable, the most relevant variables were household income per capita, PC1 and PC2, LDL-HDL-c ratio, TGL, non-HDL-c, and waist circumference (Figure 3).

**Figure 4:**
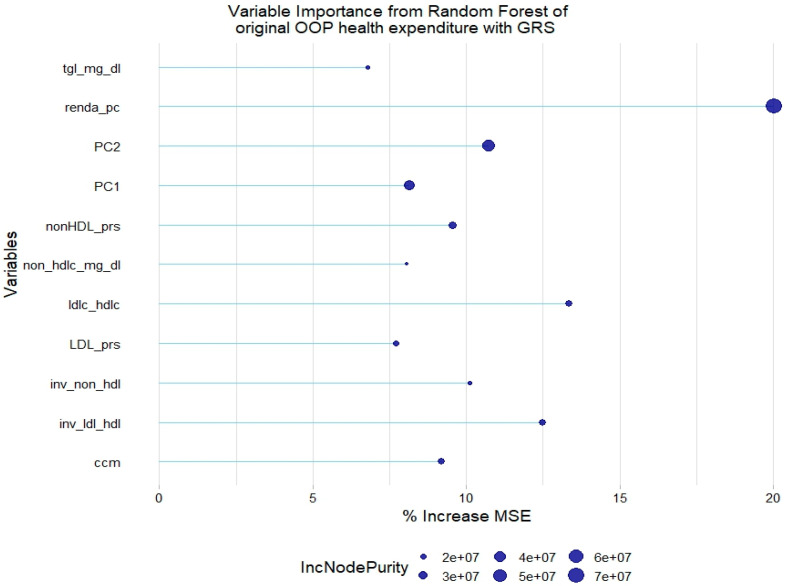
Relevant variables with more than 5% increase in mean squared error (MSE) in a random forest of original health expenditure with lipid genetic risk scores (GRSs). prs = genetic risk score; tgl = triglycerides; ldl-c = low-density lipoprotein cholesterol; hdl-c = high-density lipoprotein cholesterol; ldl_hdl = ldl-c/hdl-c ratio; PC = principal component of ancestry; renda_pc = household income; ccm = waist circumference; inv = normal-inverse-transformed version of the variable.

There was a slight increase in the absolute % of variance explanation when adding the lipid GRSs to this model. The LDL-c and non-HDL-c GRSs were relevant to explain the original health expenditure, along with the other covariates that were found to be already relevant (Figure 4).

**Figure 5:**
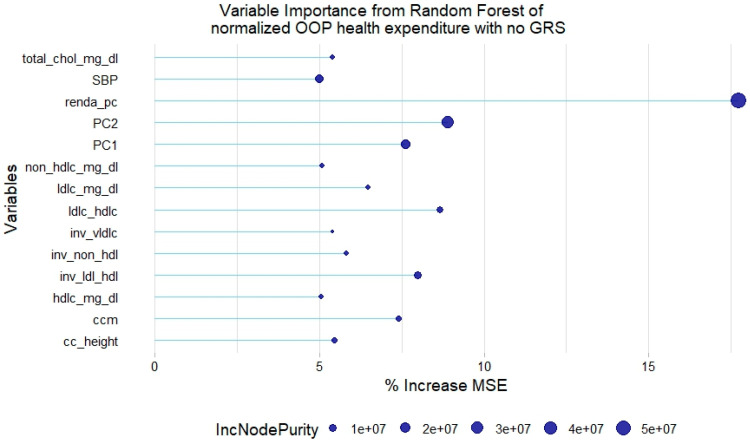
Relevant variables with more than 5% increase in mean squared error (MSE) in a random forest of normalized health expenditure without lipid genetic risk scores (GRSs). SBP = systolic blood pressure; ldl-c = low-density lipoprotein cholesterol; hdl-c = high-density lipoprotein cholesterol; vldl-c = very-low-density lipoprotein cholesterol; ldl_hdl = ldl-c/hdl-c ratio; PC = principal component of ancestry; renda_pc = household income; ccm = waist circumference; cc_height = waist circumference/height ratio; inv = normal-inverse-transformed version of the variable.

**Figure 6:**
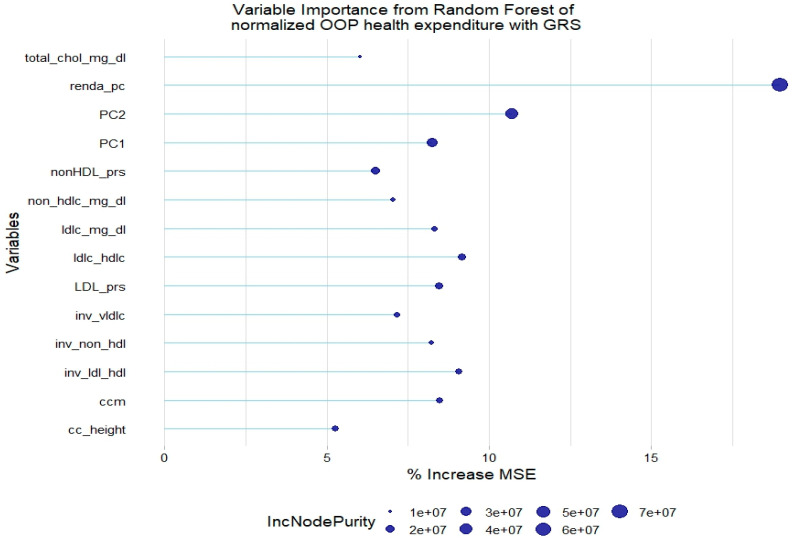
Relevant variables with more than 5% increase in mean squared error (MSE) in a random forest of normalized health expenditure with lipid genetic risk scores (GRSs). prs = genetic risk score; ldl-c = low-density lipoprotein cholesterol; hdl-c = high-density lipoprotein cholesterol; vldl-c = very-low-density lipoprotein cholesterol; ldl_hdl = ldl-c/hdl-c ratio; PC = principal component of ancestry; renda_pc = household income; ccm = waist circumference; cc_height = waist circumference/height ratio; inv = normal-inverse-transformed version of the variable.

For the normalized health expenditure outcome, all lipid traits were relevant except TGL. In addition, SBP and waist circumference/height ratio were also relevant in the model without GRSs. As with the model of original health expenditure, only LDL-c GRSs and non-HDL-c GRSs were relevant in the model with genetic scores (Figure 5 and Figure 6).

### 3.3. Categorical OOP Health Expenditures

**Figure 7:**
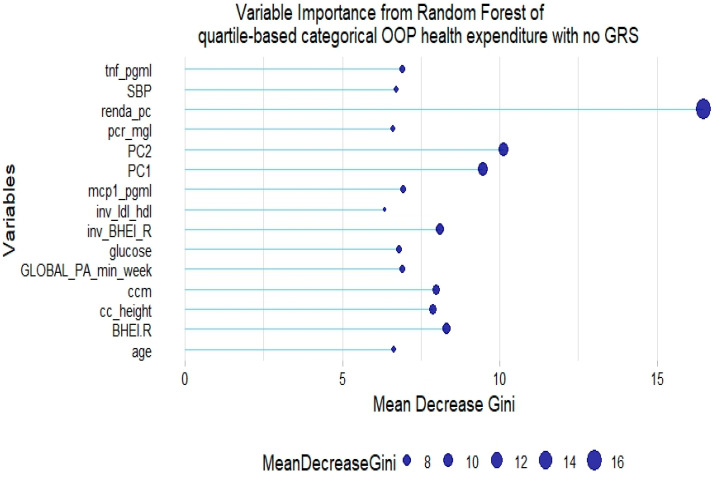
Fifteen most relevant variables according to mean decrease in the Gini index in a random forest of quartile-based categorical health expenditure without lipid genetic risk scores (GRSs). tnf = tumor necrosis factor α; pcr = C-reactive protein; BHEI-R = Brazilian revised healthy index; GLOBAL PA = global physical activity; ldl_hdl = ldl-c/hdl-c ratio; PC = principal component of ancestry; renda_pc = household income; ccm = waist circumference; cc_height = waist circumference/height ratio; inv = normal-inverse-transformed version of the variable.

The RF model of quartile-based categorical health expenditure without GRSs showed that some inflammatory markers, glucose, lifestyle factors such as diet quality and physical activity, and age are also relevant to explain the response variable (Figure 7).

**Figure 8:**
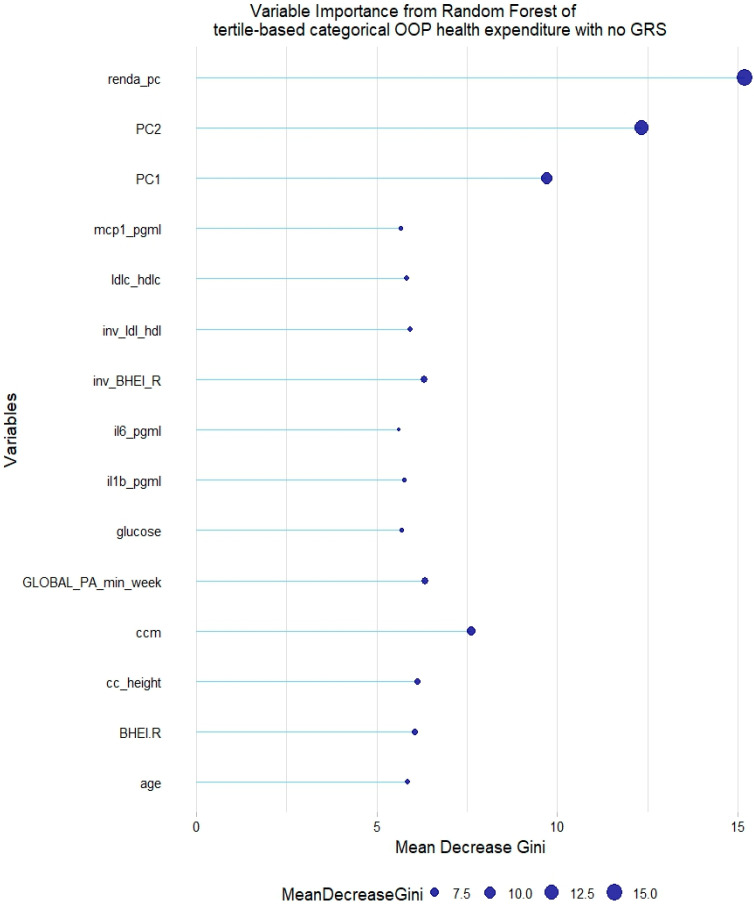
Fifteen most relevant variables according to mean decrease in the Gini index in a random forest of tertile-based categorical health expenditure without lipid genetic risk scores (GRSs). mcp1 = monocyte chemoattractant protein 1; il6 = interleukin 6; il1b = interleukin 1 β; BHEI-R = Brazilian revised healthy index; GLOBAL PA = global physical activity; ldl-c = low-density lipoprotein cholesterol; hdl-c = high-density lipoprotein cholesterol; ldl_hdl = ldl-c/hdl-c ratio; PC = principal component of ancestry; renda_pc = household income; ccm = waist circumference; cc_height = waist circumference/height ratio; inv = normal-inverse-transformed version of the variable.

**Figure 9:**
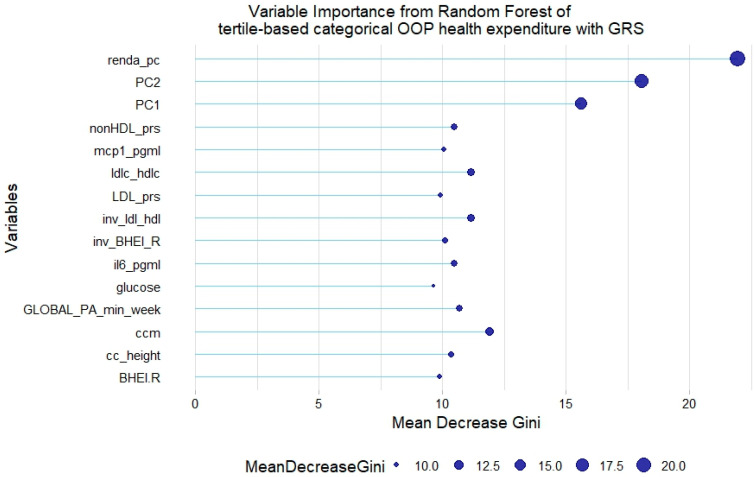
Fifteen most relevant variables according to mean decrease in the Gini index in a random forest of tertile-based categorical health expenditure with lipid genetic risk scores (GRSs). prs = genetic risk score; mcp1 = monocyte chemoattractant protein 1; il6 = interleukin 6; BHEI-R = Brazilian revised healthy index; GLOBAL PA = global physical activity; ldl-c = low-density lipoprotein cholesterol; hdl-c = high-density lipoprotein cholesterol; ldl_hdl = ldl-c/hdl-c ratio; PC = principal component of ancestry; renda_pc = household income per capita; ccm = waist circumference; cc_height = waist circumference/height ratio; inv = normal-inverse-transformed version of the variable.

However, no GRS was among the 15th most relevant variables when added to the model. This was not the case for tertile-based categorical health expenditure, for which both LDL-c GRS and non-HDL-c GRS were associated with the trait (Figure 8 and Figure 9).

In addition, results of logistic regression of quartile-based categorical health expenditure without including lipid GRSs are presented in Table 2, showing that the variables with the highest odds ratio significantly associated with quartile-based categorical health expenditure were income per capita, overweight, and the ratio LDL-c/HDL-c. None of the GRSs for lipid traits were significant when added to the logistic regression models that include the previously selected significant non-genetic variables.

**Figure 10:**
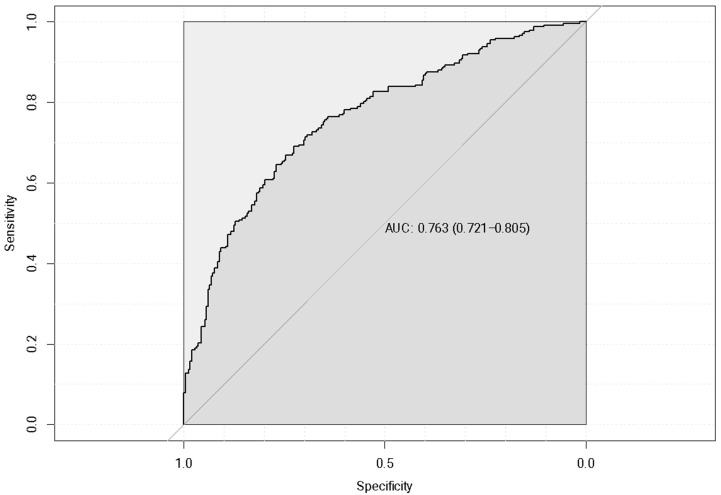
Logistic regression receiver operating characteristic (ROC) curve of quartile-based categorical health expenditure with no GWAS-derived genetic risk score (GRS) for included lipid traits.

This model had a considerably high accuracy of 0.76 as shown by its ROC curve (Figure 10).

## 4. Discussion

Despite the severe health and economic burden of dyslipidemia and related CVDs, comprehensive analyses of these and other risk factors in relation to OOP health expenditures are still scarce, especially in the context of highly admixed underrepresented populations. Hence, in the present study, we evaluated the relationship between private health expenditures and a comprehensive set of sociodemographic, cardiometabolic, anthropometric, lifestyle, and genetic risk scores related to lipid traits. The use of random forest provided significant insights into the determinants of quantitative and categorical health costs that would otherwise not be identified using traditional linear models.

Several variables were consistently relevant to explain health costs not only across different techniques such as random forest and logistic regression but also across several measures of health cost. For instance, the major non-genetic factors associated with these outcomes were household income, waist circumference, global physical activity, diet quality, inflammatory markers, and several lipid traits, especially LDL-c and LDL-c/HDL-c. The role of lifestyle in CVDs and other chronic diseases has been widely recognized and might mediate their impact on health expenditures. For instance, recent evidence from a global study estimated astonishing 499.2 million new cases of non-communicable diseases and INT$ 520 billion of direct healthcare costs, resulting from insufficient physical activity. A major part of this estimated burden would occur in low- and middle-income countries by 2030 [11]. Also, the concomitant associations of inflammatory markers, lipid phenotypes, and adiposity measures such as waist circumference with health expenditures are in line with the extensive and robust evidence of the role of inflammation as a fundamental process underlying cardiometabolic diseases and other non-communicable diseases. For instance, the accumulation of lipids in fat tissues stimulates the production of pro-inflammatory cytokines such as IL-6 and IL-1β, which can ultimately establish a chronic systemic inflammatory state [28,29,30].

Regarding healthcare expenditures, the literature on the associations between these variables and lipid traits remains quite limited, with most studies focusing either on specific dyslipidemias, such as familial hypercholesterolemia, or on evaluating the reduction in healthcare costs through lipid-lowering therapies [31,32]. Recently, Kazi et al. (2023) showed high out-of-pocket expenses pertaining to medications for patients with several CVD conditions, including hypercholesterolemia, for which the projected annual mean cost was USD 1629 [33]. An earlier study by Boudreau et al. (2009) also found a significant economic burden due to any dyslipidemia, with an additional cost estimate of USD 838 for the presence of dyslipidemia in individuals with overweight and hypertension over a long lifespan [34].

In addition to the non-genetic associations found for some lipid traits in this study, the use of lipid-related genetic risk scores, particularly the one related to non-HDL-c and LDL-c concentrations also contributed to the explanation of the phenotype of interest, as shown by the random forest technique. Furthermore, in this study, the two main principal components of ancestry were found to be strongly associated with private health expenditures across all analyses, which consistently highlights the importance of including population genetic admixture in the evaluation of health costs. This aligns with the increasingly growing recognition of the importance of studying underrepresented admixed populations in genetic research, including populations in Latin America [34,35,36]. Hence, expanding on that literature, our findings introduce the notion that an admixed genetic background could also significantly influence health cost predictions, highlighting the importance of considering genetic ancestry, by means of reducing the endogeneity bias in health expenditure predictions, thereby providing a clearer picture of the underlying determinants of health costs.

Regarding more precise genetic influences, previous investigations have also sought to unravel the effect of the genetic predisposition to cardiometabolic risk factors on health expenditures. Using longitudinal data from survey and medical claims of older adults, Wehby GL et al. (2018) found an association between a 1 SD increase in the BMI polygenic risk score and an average increase of USD 805–871 in annual health expenditures [18]. Their results were also consistent across other measures of expenditure including categorical high expenditure. Similarly, consistency among several measures of cost was present in our study, although the evaluated genetic score was not the same. In addition, even though the authors also adjusted their models for principal components of ancestry, there was no reported effect of those components on health costs, as opposed to our investigation. Noteworthily, household income and lipids traits were not included as covariates in their analysis. This omission poses a challenge for comparing these studies, especially considering other disparities in study design, population demographics, and statistical approaches.

To the best of our knowledge, this is the first study evaluating the connection between private health expenditures and lipid traits under a broad perspective, i.e., evaluating serum lipids, rather than focusing exclusively on a given dyslipidemia. Despite the complexity of analyzing multifactorial risk factors like lipids and multifactorial responses such as health costs, we successfully found significant associations of health costs with both lipid traits and their GWAS-derived genetic scores. This achievement was made possible by the use of non-traditional methods such as random forest and through the inclusion of genetic ancestry information. Moreover, the utilization of data from a highly admixed Brazilian cohort comprising free-living individuals, as a part of a population-based study representative at the population level, underscores the methodological rigor employed in data collection and analysis, which is another fundamental strength of the study.

Nonetheless, some limitations should be recognized. First, there was a limited sample size available for the analysis mainly due to a relatively high number of missing data across phenotypic and genotypic information. Second, the interpretability of the random forest results is lower than the one for traditional linear regression models in terms of the direction of effect sizes. Third, the GWAS-derived genetic risk scores may bring previous biases from the limited number of SNPs used in the association tests with their respective phenotypes (in comparison to millions of SNPs in larger GWASs), as well as other statistical limitations in those previous analyses. However, in spite of our GWAS-derived scores being estimated from a limited number of SNPs, their correlation with lipid traits was high in comparison to other findings from individuals with familial hypercholesterolemia in another Brazilian cohort from São Paulo [37]. Considering the growing recognition of SNPs and polygenic scores as useful instrumental variables to clarify the relationship of exposures and outcomes of interest of both biological and socioeconomic dimensions [38,39], our findings support the use of this genetic information as an instrument to predict other phenotypes, including health expenditures.

Further studies using data from other Brazilian admixed cohorts should be based on more precise data assessments with larger sample sizes and larger panels of SNPs. This would dramatically enhance the analytical power of the random forest models and could facilitate the use of other approaches to bring interpretability to the findings. In addition, the inclusion of more extensive SNP panels and larger and more diverse cohorts would not only enhance our understanding of the economic burden associated with dyslipidemia and CVDs, but also facilitate the integration of genetic data into public health strategies.

## 5. Conclusions

To the best of our knowledge, this is the first study performed in a highly admixed population providing relevant insights on a set of comprehensive determinants of private health expenditures. These determinants mainly comprised lifestyle, inflammatory markers, principal components of ancestry, lipid profiles, and GWAS-derived genetic risk scores related to lipid traits.

The initial hypothesis that the lipid profile and its underlying genetic background might have an impact on private health expenditures was confirmed through a machine learning approach that has not been much used in the field, highlighting its relevance to answer economics–medicine research questions. Importantly, we provided a novel view about the role of genetic ancestry on cardiometabolic risk factors and its potential impact on health costs, considering that ancestry information was among the main factors contributing to health expenses.

Further investigations with more ethnically diverse cohorts and larger data sets in terms of sample size and genetic and non-genetic information should be performed. This may aid in unraveling the role of each group of these factors in health expenses more precisely, as well as in clarifying how they might interact with each other in a mechanistic way. Finding the most suitable factors that can be targeted by public policies and clinical interventions will then be fundamental to decrease the economic burden resulting from cardiovascular diseases.

## Figures and Tables

**Table 1:** Descriptive statistics of the ISA 2015 data set for modeling analysis of OOP health expenditures.

Characteristic	Total N = 490 *
BHEI-R	66 (61, 71)
CRP (mg/L)	0.29 (0.10, 0.78)
TNF-α (pg/mL)	11.1 (8.2, 14.3)
MCP1 (pg/mL)	278 (214, 345)
IL6 (pg/mL)	1 (1, 4)
IL1β (pg/mL)	1.18 (0.91, 1.52)
IL10 (pg/mL)	4.3 (3.1, 7.0)
Overweight	
No	268 (55%)
Yes	222 (45%)
Waist circumference (cm)	91 (78, 103)
Waist circumference/height	0.56 (0.47, 0.63)
SBP	125 (115, 140)
DBP	76 (68, 83)
Ln health expenditure	5.0 (−6.9, 6.4)
Health expenditure (BRL)	154 (0, 608)
Education score	0.75 (0.38, 1.00)
Household income (BRL)	828 (521, 1536)
Hypolipidemic drug use	
No	449 (92%)
Yes	41 (8.4%)
Ethnicity	
Yellow	1 (0.2%)
White	265 (54%)
Indigenous	1 (0.2%)
Other	22 (4.5%)
Brown (Pardo)	154 (31%)
Black	47 (9.6%)
Age group	
Adolescent	145 (30%)
Adult	163 (33%)
Older adult	182 (37%)
Alcohol use	
No	361 (74%)
Yes	129 (26%)
Smoking	
Former smoker	90 (18%)
Smoker	58 (12%)
Never	342 (70%)
TGL (mg/dL)	100 (74, 143)
Total cholesterol (mg/dL)	169 (140, 200)
Non-HDL-c (mg/dL)	123 (96, 155)
LDLc (mg/dL)	101 (78, 126)
HDL-c (mg/dL)	43 (35, 53)
VLDLc (mg/dL)	20 (15, 29)
LDL-c/HDL-c	2.41 (1.66, 3.21)
DLP adj.	
No	168 (34%)
Yes	322 (66%)
PC1	−0.003 (−0.009, 0.002)
PC2	−0.013 (−0.018, −0.010)
Sex	
Female	225 (46%)
Male	265 (54%)
Age (years)	49 (18, 64)
Diabetes	
No	415 (85%)
Yes	75 (15%)
HOMA-IR	2.79 (1.82, 4.25)
Insulin resistance	
No	247 (50%)
Yes	243 (50%)
Insulin (µUI/mL)	11 (8, 17)
Glucose (mg/dL)	95 (88, 105)
Global PA (min/week)	440 (170, 1154)
Leisure PA (min/week)	0 (0, 135)
Quartile-based health expenditure	
Low	248 (51%)
High	242 (49%)
Tertile-based health expenditure	
Low	330 (67%)
High	160 (33%)
Normalized health expenditure	154 (0, 608)
Inv total cholesterol	−0.01 (−0.70, 0.67)
Inv TGL	0.00 (−0.67, 0.68)
Inv HDL-c	0.00 (−0.67, 0.69)
Inv LDL-c	−0.02 (−0.67, 0.65)
Inv LDL-c/HDL-c	−0.01 (−0.66, 0.66)
Inv VLDL-c	0.00 (−0.64, 0.70)
Inv non-HDL-c	−0.02 (−0.66, 0.66)
Inv BHEI-R	−0.03 (−0.69, 0.65)
Log10 health expenditure	2.19 (−3.00, 2.78)
HDL-c GRS	
−0.555	41 (8.4%)
−0.277	177 (36%)
0.000	224 (46%)
0.004	5 (1.0%)
0.282	20 (4.1%)
0.559	23 (4.7%)
LDL-c/HDL-c GRS	
0.000	218 (44%)
0.251	206 (42%)
0.502	66 (13%)
LDL-c GRS	0.02 (−0.40, 0.47)
Non-HDL-c GRS	0.23 (−0.17, 0.59)
TGL GRS	
0.000	375 (77%)
0.392	47 (9.6%)
0.441	50 (10%)
0.785	7 (1.4%)
0.834	10 (2.0%)
1.275	1 (0.2%)
Total cholesterol GRS	0.00 (−0.25, 0.29)
VLDL-c GRS	
0.000	425 (87%)
0.405	58 (12%)
0.811	7 (1.4%)

* Median (IQR = interquartile range); N (%) (absolute frequency and proportion); TGL = triglycerides; LDL-c = low-density lipoprotein cholesterol; HDL-c = high-density lipoprotein cholesterol; VLDL-c = very-low-density lipoprotein cholesterol; PC = principal component of ancestry; MCP1 = monocyte chemoattractant protein; CRP = C-reactive protein; TNF-α = tumor necrosis factor α; SBP = systolic blood pressure; DBP = diastolic blood pressure; global PA = global physical activity; leisure PA = leisure physical activity; HOMA-IR = homeostatic model assessment for insulin resistance; BHEI-R = revised Brazilian healthy eating index; DLP adj. = any dyslipidemia adjusted by hypolipidemic drug; GRS = genetic risk score; Inv = normal-inverse transformed.

**Table 2:** Significant associations between quartile-based categorical health expenditure with lipid traits and other non-genetic variables.

Variable	Estimate	Std. Error	z Value	*p*-Value	Odds Ratio	Lower CI	Upper CI
Overweight (Yes/No)	0.558	0.235	2.377	0.017	1.747	1.106	2.780
Income per capita	0.001	0.000	5.015	0.000	1.001	1.000	1.001
LDL-c (mg/dL)	−0.083	0.029	−2.864	0.004	0.920	0.867	0.971
LDL-c/HDL-c	1.075	0.371	2.898	0.004	2.930	1.453	6.237
PC2	−43.889	16.500	−2.660	0.008	0.000	0.000	0.000
Age (years)	0.014	0.007	2.066	0.039	1.014	1.001	1.028
Glucose (mg/dL)	0.006	0.003	2.107	0.035	1.006	1.001	1.013
Inv LDL-c	5.596	1.852	3.022	0.003	269.241	8.324	11,972.248
Inv LDL-c/HDL-c	−2.828	1.011	−2.796	0.005	0.059	0.007	0.381

Obs: LDL-c—low-density lipoprotein cholesterol; HDL-c—high-density lipoprotein cholesterol; inv—inverse-normal-transformed trait; and PC—principal component of ancestry.

## Data Availability

The data presented in this study are available on request from the corresponding author due to privacy and institutional restrictions.

## Supplementary Materials

The following supporting information can be downloaded at: https://www.mdpi.com/article/10.3390/healthcare12222275/s1, Table S1. Composition of Genetic Risk Scores with SNPs significantly associated with lipid traits.
